# Supplementary figures and images for: MARCH family E3 ubiquitin ligases selectively target and degrade cadherin family proteins
Source: PLoS One. 2024 May 9;19(5):e0290485. doi: 10.1371/journal.pone.0290485 (PMC11081302; doi:10.1371/journal.pone.0290485)

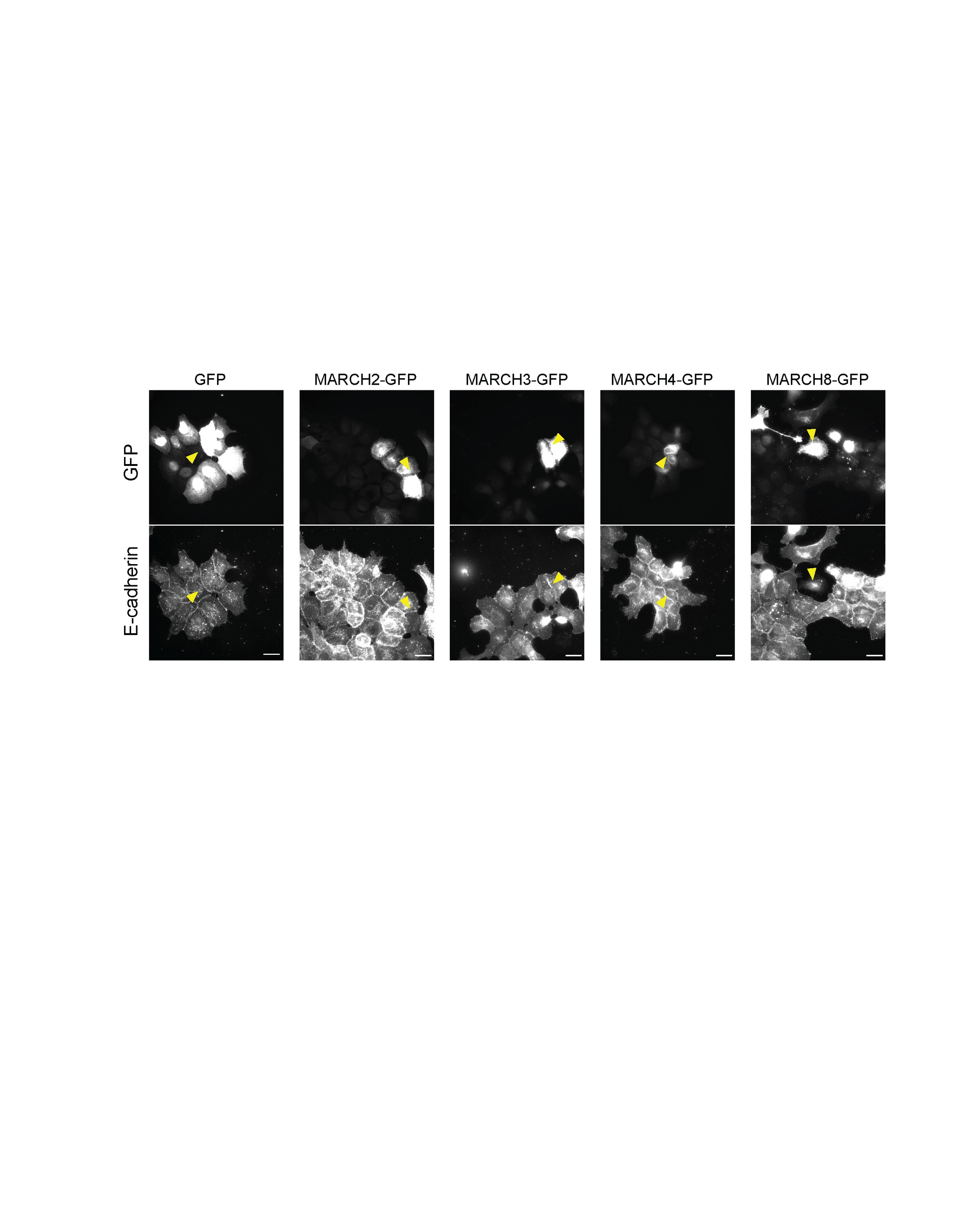

Supplement: S1 Fig — A431 cells were transfected with MARCH family proteins and subjected to immunofluorescence with E-cad antibody then processed for fluorescence microscopy. Transfected cells are indicated with yellow arrowhead. Scale bar = 25 μm. (TIF) [file pone.0290485.s001.tif]

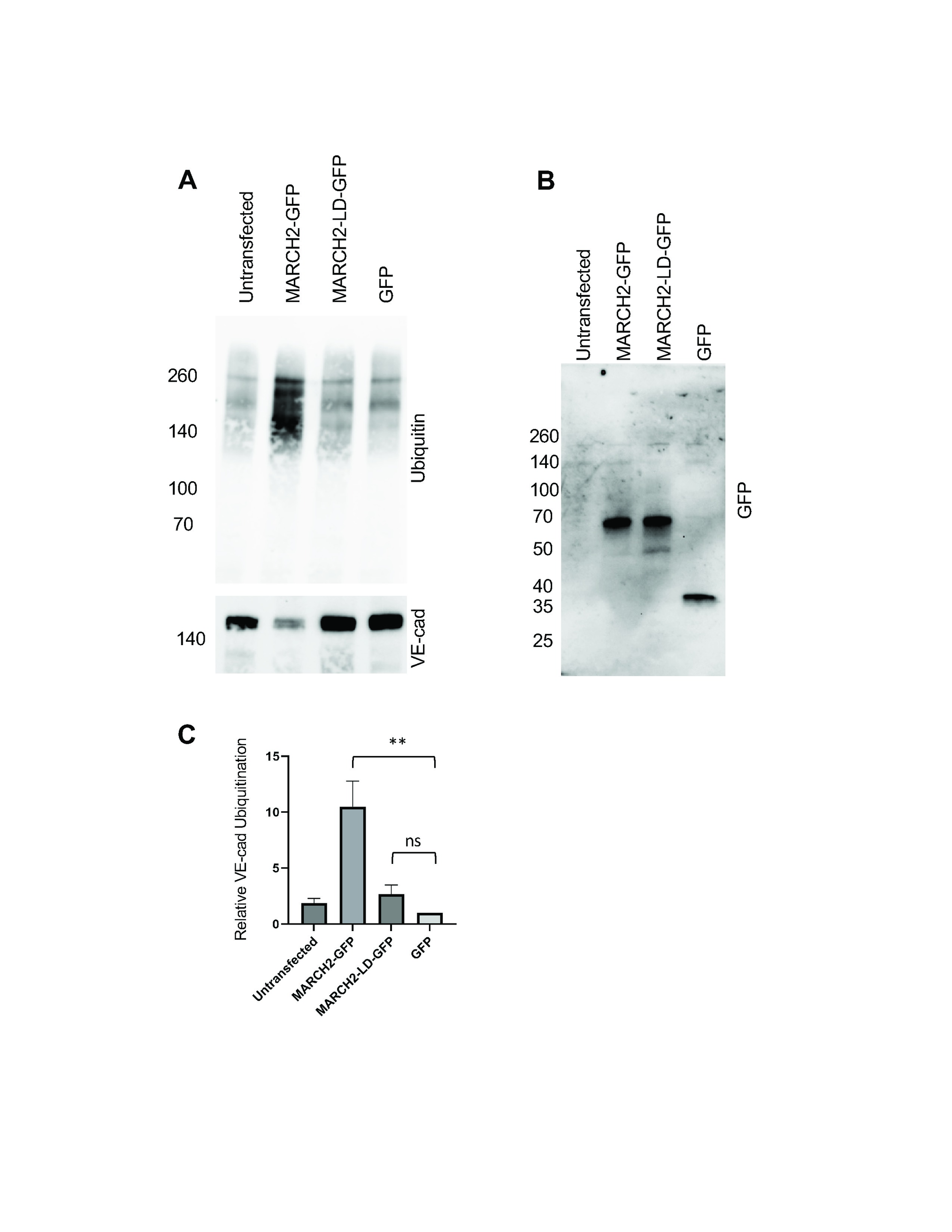

Supplement: S2 Fig — Western blot analysis of A431 cells expressing VE-cadherin. MARCH2-GFP, MARCH2-LD-GFP or GFP were expressed using adenoviral delivery systems. Cell lysates were subjected to immunoprecipitation using a VE-cadherin antibody followed by western blot directed against ubiquitin or VE-cadherin (Panel A). Similar expression levels of GFP, MARCH2-GFP or MARCH2-LD-GFP were verified by western blot of whole cell lysates using antibodies directed against GFP (Panel B). Quantitative assessment of ubiquitinated VE-cadherin is shown in Panel C. Relative ubiquitination level was measured by densitometry and normalized to the level of VE-cadherin immunoprecipitated. Data represent the mean and SEM from 3 independently conducted experiments (**p<0.01, ns = not significant). (TIF) [file pone.0290485.s002.tif]

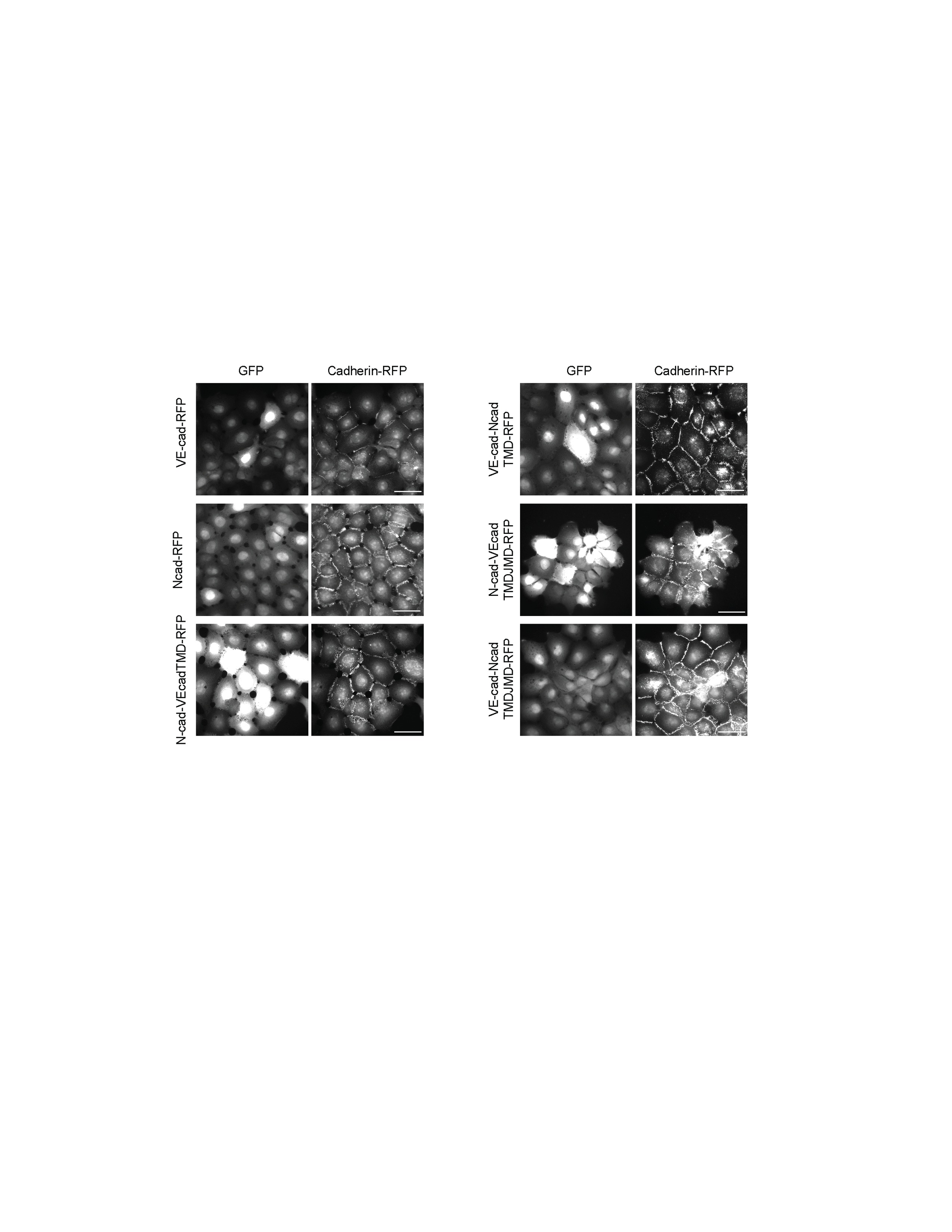

Supplement: S3 Fig — Localization of RFP tagged various cadherin chimeras were assessed in A431 cells expressing GFP. Scale bar = 50 μm. (TIF) [file pone.0290485.s003.tif]

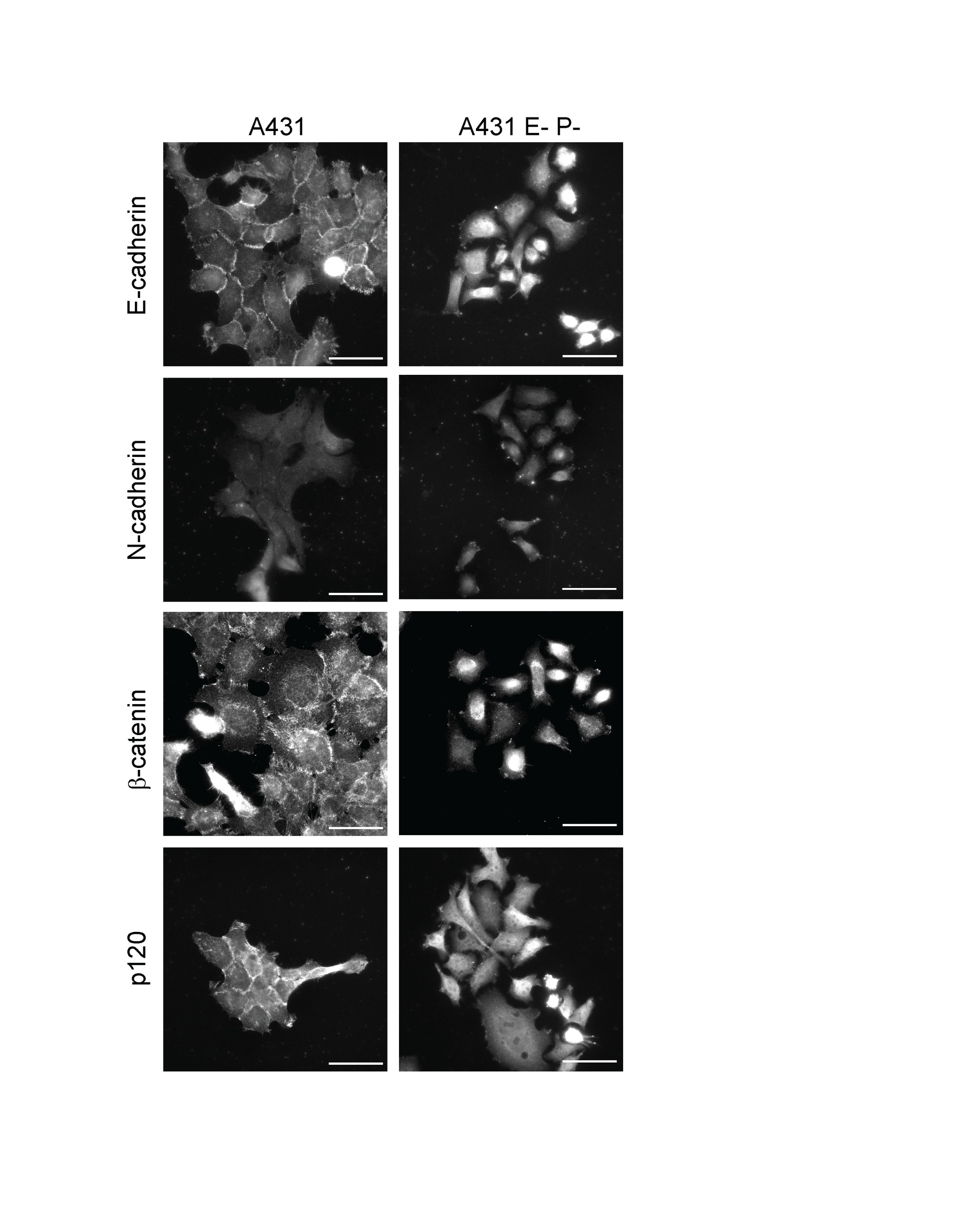

Supplement: S4 Fig — Wild type A431 cells or A431 cells lacking E- and P-cadherin [43] were processed for fluorescence microscopy to localize E-cadherin, N-cadherin, β-catenin or p120-catenin. The lack of β-catenin and p120-catenin at cell-cell contacts confirms lack of other classical cadherins. Scale bar = 50 μm. (TIF) [file pone.0290485.s004.tif]

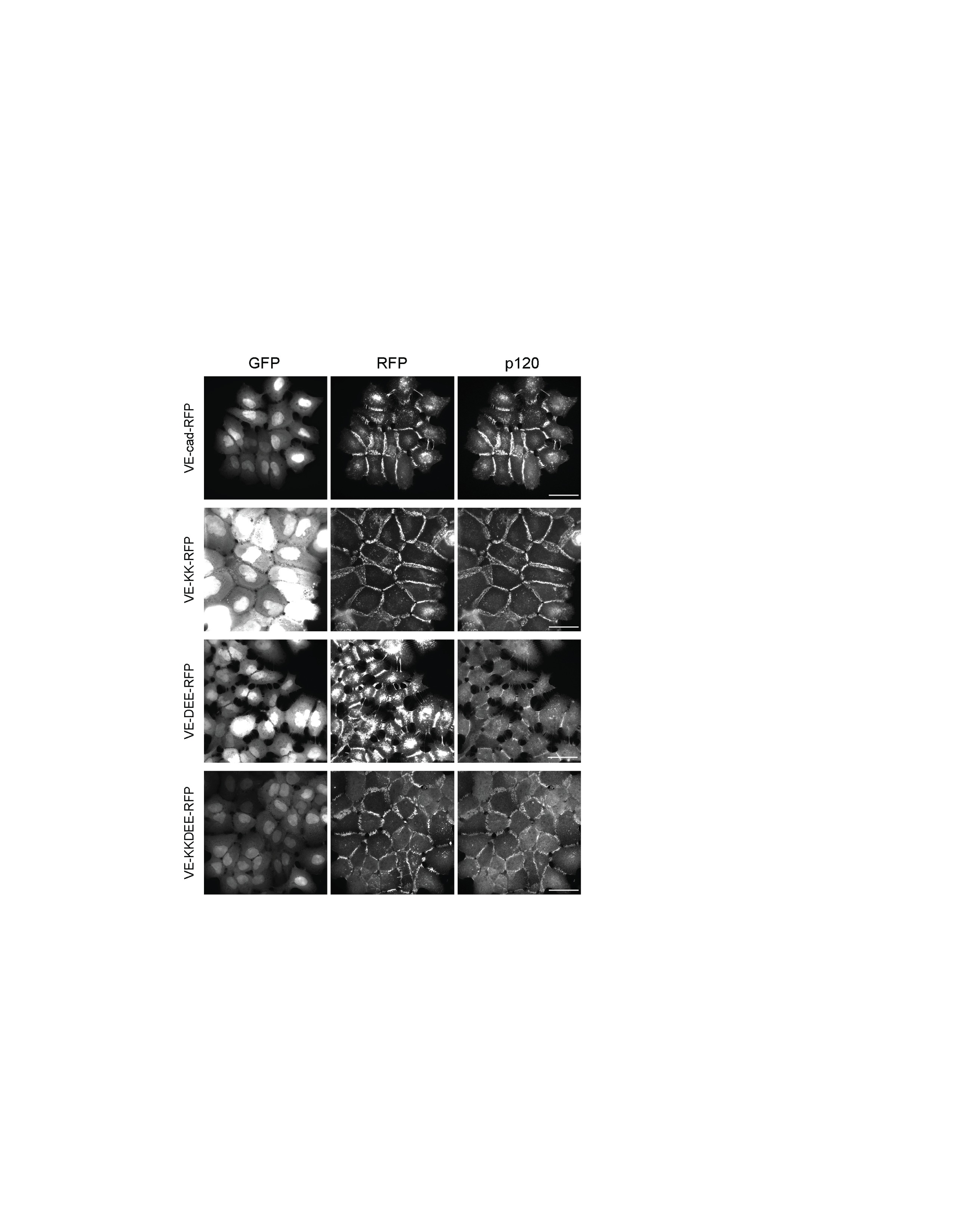

Supplement: S5 Fig — E-cadherin/P-cadherin null A431 cells expressing various VE-cadherin mutants were analyzed by immunofluorescence for localization of the cadherin and p120-catenin in cells expressing GFP. Scale bar = 25 μm. (TIF) [file pone.0290485.s005.tif]
