# Supplementary material for: MARCH family E3 ubiquitin ligases selectively target and degrade cadherin family proteins
Source: PLoS One. 2024 May 9;19(5):e0290485. doi: 10.1371/journal.pone.0290485 (PMC11081302; doi:10.1371/journal.pone.0290485)
Supplement: S1 Raw image — (PDF) [file pone.0290485.s006.pdf]

Figure S2 (VE-cad)

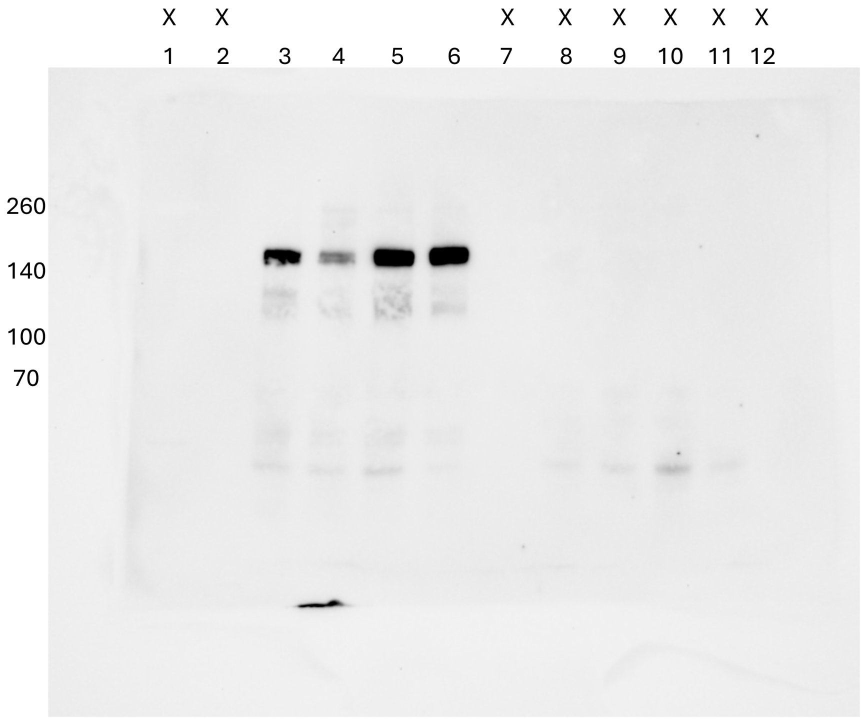

1	Marker
2	1xsample buffer
3	untransfected Beads fraction
4	MARCH2-GFP Beads fraction
5	MARCH2-LD-GFP Beads fraction
6	GFP Beads fraction
7	1x sample buffer
8	untransfected no Antibody Beads fraction
9	MARCH2-GFP no Antibody Beads fraction
10	MARCH2-LD-GFP no Antibody Beads fraction
11	GFP no Antibody Beads fraction
12	1x sample buffer

Membrane was developed by ECL and fluorescence through a 647 SP emission filter was captured

Figure S2 (ubiquitin)

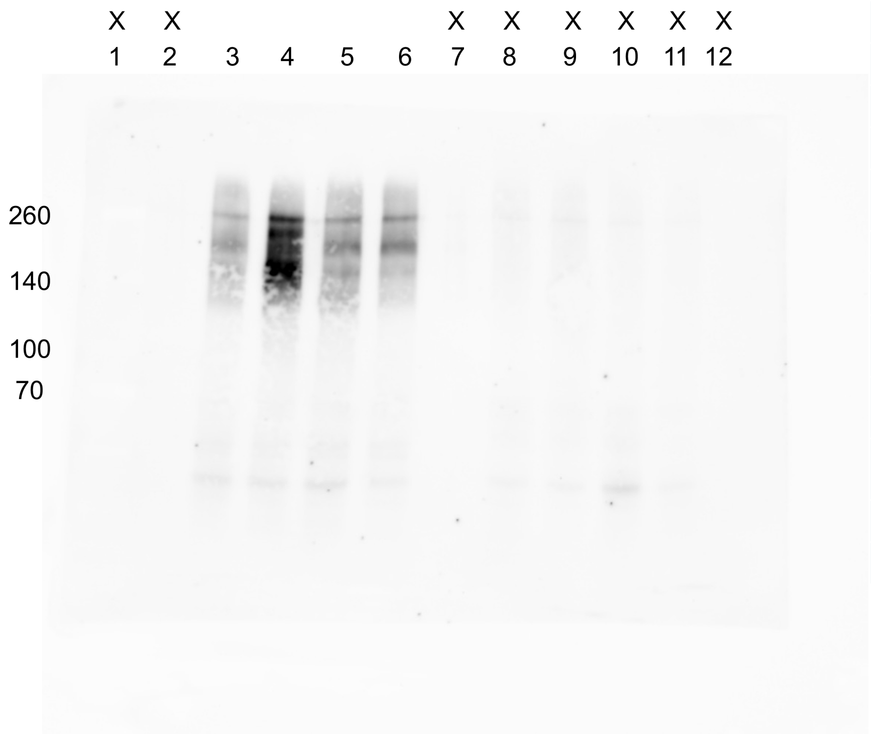

1	Marker
2	1xsample buffer
3	untransfected Beads fraction
4	MARCH2-GFP Beads fraction
5	MARCH2-LD-GFP Beads fraction
6	GFP Beads fraction
7	1x sample buffer
8	untransfected no Antibody Beads fraction
9	MARCH2-GFP no Antibody Beads fraction
10	MARCH2-LD-GFP no Antibody Beads fraction
11	GFP no Antibody Beads fraction
12	1x sample buffer

Membrane was developed by ECL and fluorescence through a 647 SP emission filter was captured

Figure S2 (GFP)

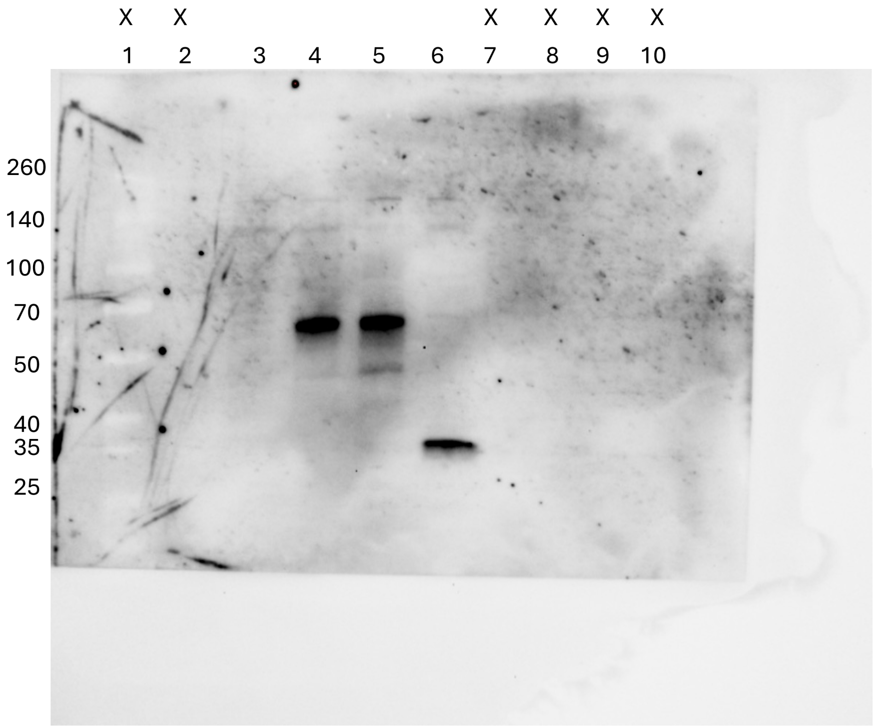

1	Marker
2	1xSample buffer
3	Untransfected input
4	MARCH2-GFP input
5	MARCH2-LD-GFP input
6	GFP input
7	1xSample buffer
8	blank
9	blank
10	blank

Membrane was developed by ECL and fluorescence through a 647 SP emission filter was captured

Figure 4B (actin)

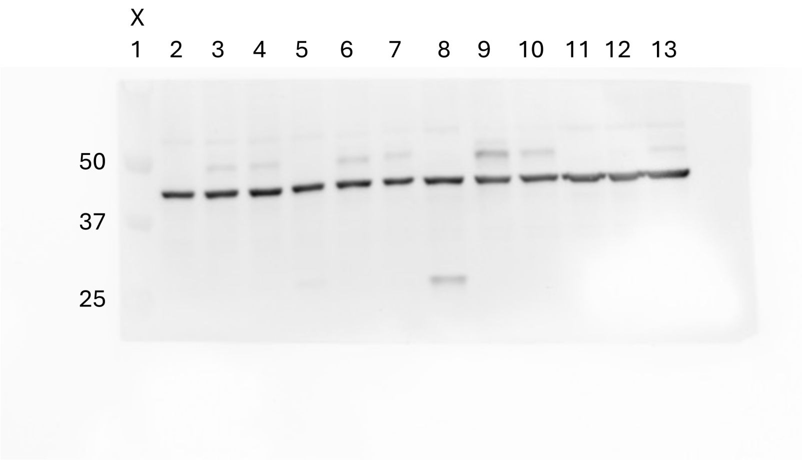

1	Marker
2	VE-WT with GFP
3	VE-WT with MARCH2-LD-GFP
4	VE-WT with MARCH2-GFP
5	VE-KK with GFP
6	VE-KK with MARCH2-LD-GFP
7	VE-KK with MARCH2-GFP
8	VE-DEE with GFP
9	VE-DEE with MARCH2-LD-GFP
10	VE-DEE with MARCH2-GFP
11	VE-KKDEE with GFP
12	VE-KKDEE with MARCH2-LD-GFP
13	VE-KKDEE with MARCH2-GFP

Membrane was developed by ECL and fluorescence through a 647 SP emission filter was captured

Figure 4B (VE-cadherin and GFP)

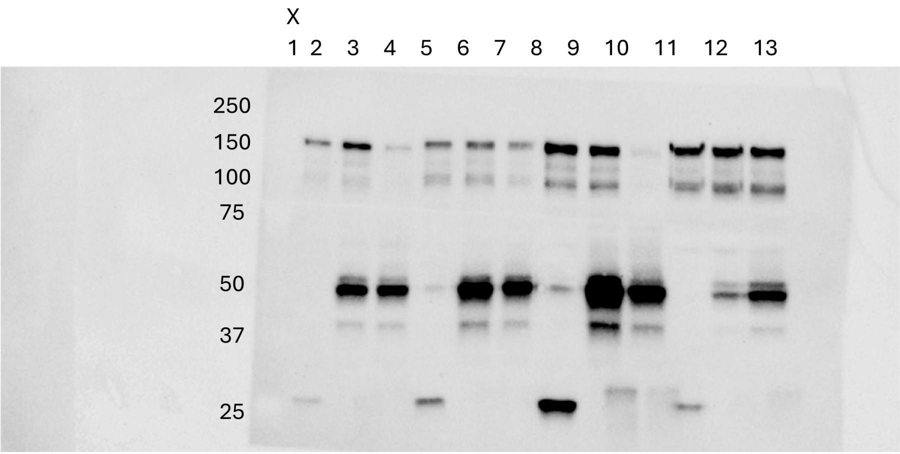

1	Marker
2	VE-WT with GFP
3	VE-WT with MARCH2-LD-GFP
4	VE-WT with MARCH2-GFP
5	VE-KK with GFP
6	VE-KK with MARCH2-LD-GFP
7	VE-KK with MARCH2-GFP
8	VE-DEE with GFP
9	VE-DEE with MARCH2-LD-GFP
10	VE-DEE with MARCH2-GFP
11	VE-KKDEE with GFP
12	VE-KKDEE with MARCH2-LD-GFP
13	VE-KKDEE with MARCH2-GFP

Membrane cut under 100 kDa. Upper membrane incubated with VE-cadherin antibody. Lower membrane incubated with GFP antibody.
Membrane was developed by ECL and fluorescence through a 647 SP emission filter was captured

Fig2C upper (GFP)

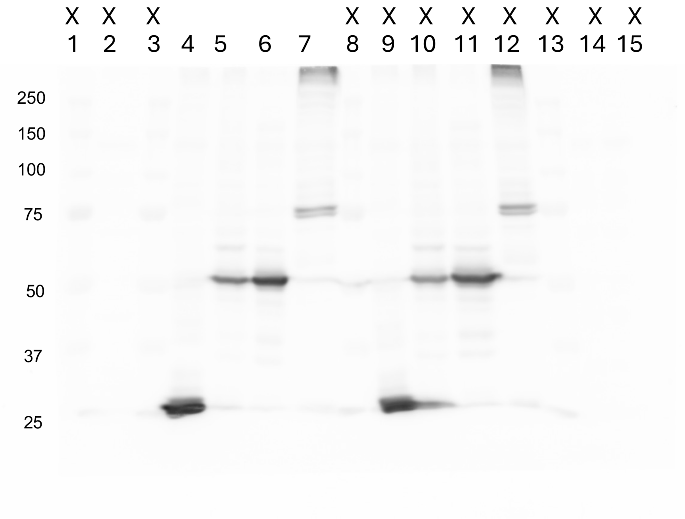

1	Marker
2	Untransfected
3	Marker
4	GFP
5	MARCH2-GFP
6	MARCH2-LD-GFP
7	MARCH4-GFP
8	Marker
9	GFP
10	MARCH2-GFP
11	MARCH2-LD-GFP
12	MARCH4-GFP
13	Marker
14	Filler lysate
15	Filler lysate

Membrane was excited blue epi illumination and fluorescence through a 715/30 emission filter was captured

Fig2C upper (actin)

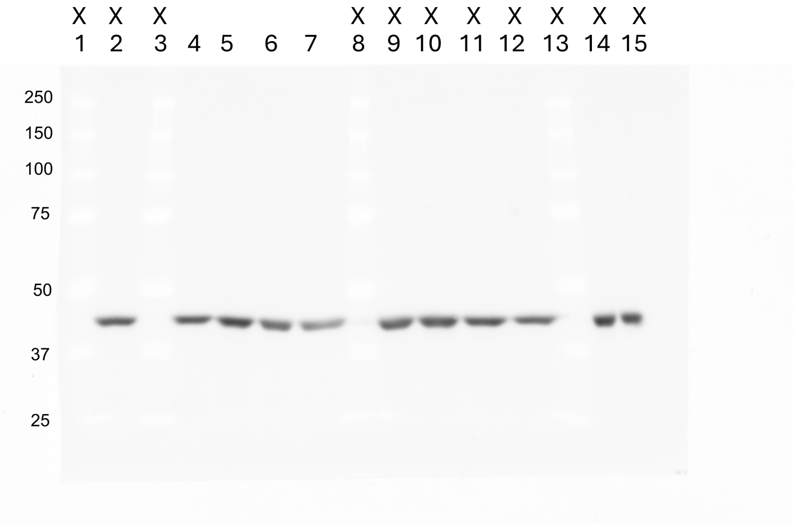

1	Marker
2	Untransfected
3	Marker
4	GFP
5	MARCH2-GFP
6	MARCH2-LD-GFP
7	MARCH4-GFP
8	Marker
9	GFP
10	MARCH2-GFP
11	MARCH2-LD-GFP
12	MARCH4-GFP
13	Marker
14	Filler lysate
15	Filler lysate

Membrane was excited green epi illumination and fluorescence through a 602/50 emission filter was captured

Fig2C upper (VE-cadherin)

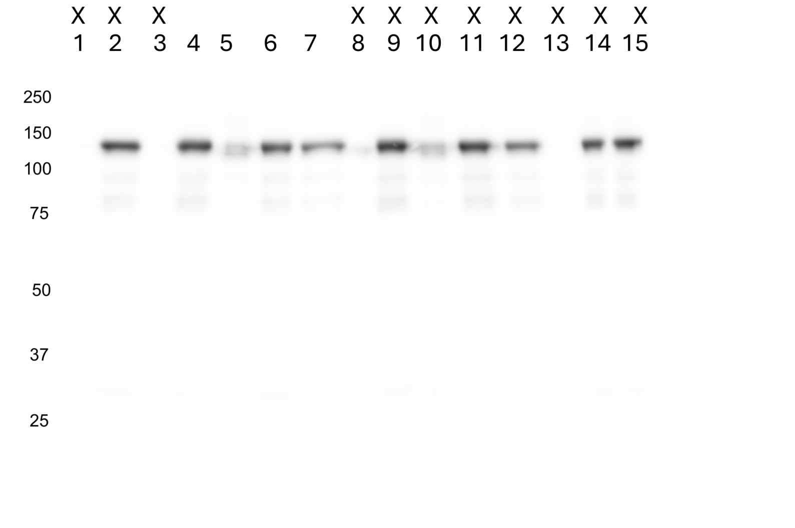

1	Marker
2	Untransfected
3	Marker
4	GFP
5	MARCH2-GFP
6	MARCH2-LD-GFP
7	MARCH4-GFP
8	Marker
9	GFP
10	MARCH2-GFP
11	MARCH2-LD-GFP
12	MARCH4-GFP
13	Marker
14	Filler lysate
15	Filler lysate

Membrane was developed by ECL and fluorescence through a 647 SP emission filter was captured

Fig2C lower (actin)

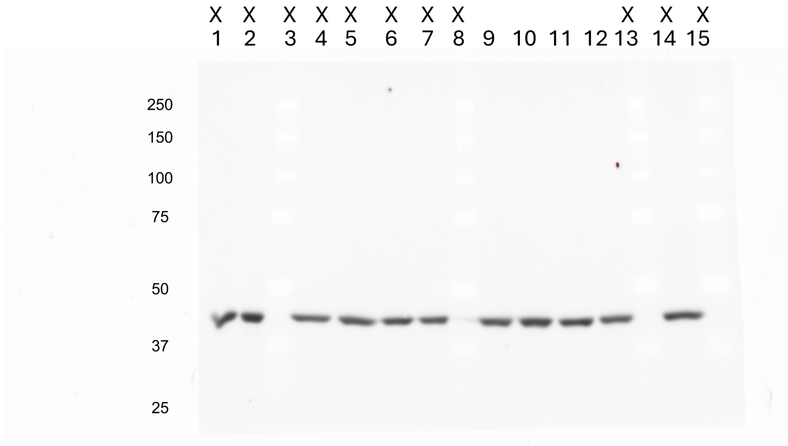

1	Filler lysate
2	Filler lysate
3	Marker
4	GFP
5	MARCH2-GFP
6	MARCH2-LD-GFP
7	MARCH4-GFP
8	Marker
9	GFP
10	MARCH2-GFP
11	MARCH2-LD-GFP
12	MARCH4-GFP
13	Marker
14	Marker
15	Untransfected

Membrane was excited green epi illumination and fluorescence through a 602/50 emission filter was captured

Fig2C lower (VEGFR2)

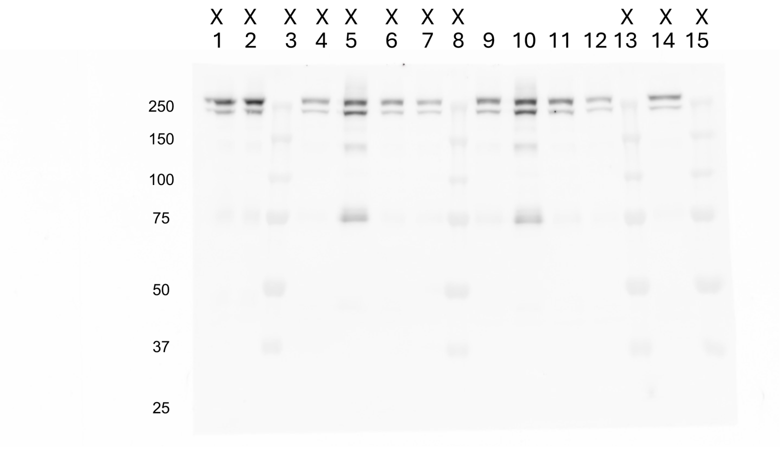

1	Filler lysate
2	Filler lysate
3	Marker
4	GFP
5	MARCH2-GFP
6	MARCH2-LD-GFP
7	MARCH4-GFP
8	Marker
9	GFP
10	MARCH2-GFP
11	MARCH2-LD-GFP
12	MARCH4-GFP
13	Marker
14	Marker
15	Untransfected

Membrane was excited blue epi illumination and fluorescence through a 715/30 emission filter was captured

Fig2C lower (N-cadherin)

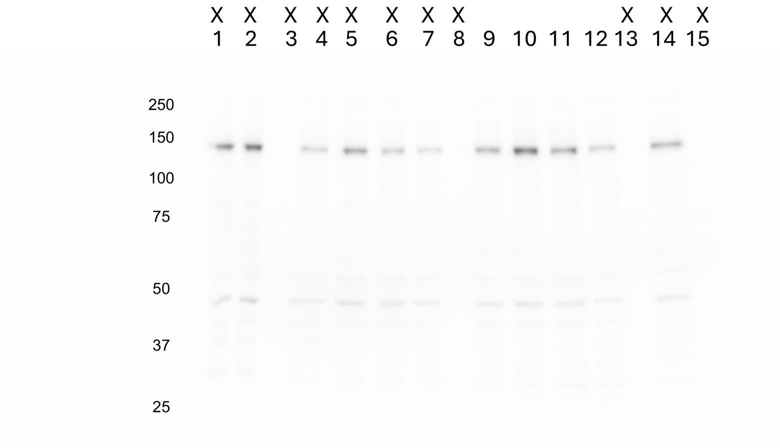

1	Filler lysate
2	Filler lysate
3	Marker
4	GFP
5	MARCH2-GFP
6	MARCH2-LD-GFP
7	MARCH4-GFP
8	Marker
9	GFP
10	MARCH2-GFP
11	MARCH2-LD-GFP
12	MARCH4-GFP
13	Marker
14	Marker
15	Untransfected

Membrane was developed by ECL and fluorescence through a 647 SP emission filter was captured
